# Domestic, family and sexual violence polyvictimisation and health experiences of Australian nurses, midwives and carers: a cross-sectional study

**DOI:** 10.1186/s12889-024-19680-7

**Published:** 2024-08-22

**Authors:** Elizabeth Veronica-Mary McLindon, Anneliese Spiteri-Staines, Kelsey Hegarty

**Affiliations:** 1 The University of Melbourne, Melbourne, Australia; 2 The Royal Women’s Hospital, Melbourne, Australia

**Keywords:** Intimate partner violence, Domestic, Family and sexual violence, Child abuse, Nursing, Health professionals, Polyvictimisation, Health associations, Well-being

## Abstract

**Background:**

Domestic, family and sexual violence is a prevalent health and social issue. Nurses may be exposed to higher rates of this violence in their personal lives compared to the community, but little is known about their polyvictimisation experiences or health and well-being impacts.

**Methods:**

An online descriptive, cross-sectional survey of women nurses, midwives and carer members of the Australian Nursing and Midwifery Federation (ANMF) (Victorian Branch) (response rate: 15.2% of nurses sent an invitation email/28.4% opened the email). Violence survey measures included: intimate partner violence (Composite Abuse Scale); child abuse and sexual violence (Australian Bureau of Statistics Personal Safety Survey items). Health measures included: Short Form-12; Fast Alcohol Screening Test; Patient Health Questionnaire-4; Short Screening for DSM-IV Posttraumatic Stress Disorder; well-being measures included: Connor-Davidson Resilience Scale, social support, and financial stress. Proportions were used to describe the prevalence of violence by sociodemographic characteristics and health and well-being issues; logistic regression predicted the odds of experiencing overlapping types of violence and of experiencing health and well-being outcomes.

**Results:**

5,982 participants (from a parent study of 10,674 nurses, midwives and carers) had experienced at least one type of lifetime violence; half (50.1%) had experienced two or three types (polyvictimisation). Survivors of child abuse were three times more likely to experience both intimate partner violence and non-partner adult sexual assault. Any violence was associated with poorer health and well-being, and the proportion of affected participants increased as the types of violence they had experienced increased. Violence in the last 12-months was associated with the poorest health and well-being.

**Conclusions:**

Findings suggest a cumulative, temporal and injurious life course effect of domestic, family and sexual violence. The polyvictimisation experiences and health and well-being associations reported by survivor nurses, midwives and carers underscores the need for more accessible and effective workplace interventions to prevent and mitigate psychosocial ill health, especially in the recent aftermath of violence.

## Background

Domestic, family and sexual violence (DFV) is a public health issue associated with substantial physical and psychological impacts for survivors, their families and the broader community [1]. DFV is common; 27% of Australian women have experienced physical, sexual or psychological intimate partner violence (IPV) since the age of 15 years [2], with the worldwide prevalence between 35% and 58% of women [3, 4]. Nearly one in three women (29%) have experienced sexual violence by someone [5]. Many Australian women (39%) grow up in families where there is abuse [6] and 18% report being exposed to physical or sexual abuse before the age of fifteen [2].

When an individual has experienced multiple victimisations of different types, such as child abuse, IPV or non-partner sexual violence, they are referred to as polyvictims or survivors of polyvictimisation [7]. DFV refers to behaviour within either an intimate partner or family relationship that causes physical, sexual or psychological harm [4]. Within this paper, the term DFV is used to refer to more than one type of abuse perpetrated by different people across a survivor’s life course, including IPV, non-stranger sexual assault and child abuse. IPV is violence perpetrated within an adult romantic relationship [4], and within this paper, the term is used to reflect physical, sexual or psychological violence by an intimate partner, usually within the last 12-months. While all DFV is associated with increased likelihood of reporting physical and mental health issues [8–10], survivors of polyvictimisation may experience even poorer health outcomes compared with survivors of single-type victimisation, suggesting a cumulative or dose-response association [7–9, 11]. However, few studies of the relationship between DFV and health have encompassed different types of violence [10, 12]. There is also limited evidence about the temporal effect of recent IPV on health and wellbeing; some evidence suggests an association between IPV in the previous 12-months and postpartum depression and drug use, although more research is needed [13].

DFV survivors are overrepresented among those presenting for healthcare, and healthcare professionals, the majority of whom are women, are ideally positioned to identify and respond to the health sequel of violence [14, 15]. Research has indicated that nurses, midwives, carers and other health professionals (hereafter referred to as ‘nurses’) may themselves experience a higher prevalence of violence in their homes than community prevalence rates [16–20]. Some research suggests acute and chronic multi-health consequences of DFV against nurses including injury, chronic fatigue, miscarriage, pre-term labour, sleep disturbance, depression and anxiety [21]. However, insufficient evidence about the prevalence and health impacts of all types of DFV persists, including polyvictimisation experiences for the largest health professional group: nurses.

To fill these research gaps, this study’s aim was to examine the proportion of nurse survivors who had experienced polyvictimisation, the odds of having experienced child abuse and/or non-partner adult sexual assault as a nurse survivor of IPV, and to understand the proportion and likelihood of physical, psychological health and well-being issues after one, two or three types of violence across the life course, including when IPV had occurred in the last 12-months. It was hypothesised that (1) participants who had experienced child abuse would be more likely to experience adult types of DFV; (2) the frequency, proportion and odds likelihood of health (general physical health, hazardous alcohol consumption, depression, anxiety, posttraumatic stress) and well-being issues (resilience, social support, financial stress) would increase as the types of violence to which a participant had been exposed, increased; (3) survivors whose IPV had occurred in the last 12-months would report the poorest health and well-being.

## Methods

### Participants

This study addresses experiences of 5,982 Australian nurses, midwives and carers who had experienced one or more types of DFV from a parent study of 10,674 nurses, midwives and carers (Blinded). While men also participated in the parent study, the disproportionately smaller number of men participants compared to women – while reflective of the nursing workforce - prevented men’s data from being included in the present study because their sample was not large enough to conduct reliable analyses. A cross-sectional survey about experiences of violence, health, employment and service use was sent to all members of the Australian Nursing and Midwifery Federation (ANMF) (Vic Branch) between 30 August 2019 and 7 February 2020. Informed consent to participate in this voluntary and confidential online survey was obtained from all participants through completed and returned surveys [22]. A project information email was sent by the ANMF Secretary to 70,124 women members and 27,759 opened the email containing an online survey link. A full description of the survey setting, recruitment process and survey measures has been reported in a paper about DFV prevalence [18].

### Measurement

IPV was measured using the Composite Abuse Scale (CAS), a 30-item validated self-report measure of abusive behaviours across the adult lifetime since the age of sixteen years (yes/no), and during the last 12-months (six-point frequency scale) [23]. Standard CAS cut off scores were used to determine and categorise 12-month abuse as ‘severe combined abuse’, ‘physical abuse combined with emotional abuse and/or harassment’, ‘physical abuse alone’, or ‘emotional abuse and/or harassment’ [23]. Adult lifetime IPV was defined as qualifying for 12-month abuse (any of the four categories) or an adult lifetime score on either or two CAS subscales: ‘severe combined abuse’ or ‘physical abuse combined with emotional abuse and/or harassment’ [18].

Non-partner adult sexual assault and child abuse were measured using the Personal Safety Survey definition and items [24]. Sexual assault was defined as including rape, attempted rape and other forced sexual activity since the age of fifteen years by somebody other than a partner (yes/no) [24]. Child abuse was defined as harmful behaviour of a physical (hit, beat, kicked, physical restraint) or sexual nature, occurring before the age of fifteen years, perpetrated by an adult over the age of eighteen years [24].

Single trauma survivor participants were defined as those who had experienced IPV, non-partner adult sexual assault or child abuse not in combination with another type of abuse. Polyvictimised survivor participants were defined as those who had experienced two or three types of DFV in any combination.

Eight areas of health and well-being were investigated using validated measures where standard scoring was applied [25–29] (Table 1). Two wellbeing issues (social support and financial stress) had to be developed for the purposes of this study because of a lack of available and appropriate brief measures. These items were based on previous research, were piloted and the scoring is detailed in Table 1.

**Table 1:** Health and well-being measures

Health and well-being ^a^	Measure	Number of items	Timeframe
**General health**			
Self-reported general health	SF-12 [29]	12	4 weeks
Hazardous alcohol consumption	FAST [26]	4	1 year
**Psychological health**			
Depression	PHQ-4 [27]	2 (of 4 psychological distress items)	2 weeks
Anxiety	PHQ-4 [27]	2 (of 4 psychological distress items)	2 weeks
Posttraumatic Stress (PTSD)	Short Screening Scale for DSM-IV Posttraumatic Stress Disorder [25]	7	1 month
**Well-being impacts**			
Resilience	CD-RISC2 [28]	2	Present day
Social support (family/friends)	Bespoke ^b^	1	Present day
Financial stress	Bespoke ^c^	1	12 months

*Note*^a^ All measures indicate presence of health/well-being issue but are not a clinical diagnosis; ^b^ Using a 5-point Likert-type scale ranging from ‘Not at all’ (1) to ‘True nearly all of the time’ (5), participants were asked, “I can get support from friends or family members”. Scores of ≤ 3 indicated lack of social support; ^c^ Using a 5-point Likert-type scale ranging from ‘Never in the last 12 months’ (0) to ‘Daily’ (5), participants were asked: “How often in the last 12-months have you experienced financial stress”. Scores of ≥ 2 indicated financial stress

### Analysis

Univariate analyses using frequencies and percentages determined the prevalence of single type and multi-type violence. Logistic regression analysis was used to model the association between outcome (binary) and exposure variables through odds ratios (OR) [30]. ORs, 95% confidence intervals (CI) and *P-*values were employed to assess the likely size of the association between violence and health variables. To assess for the potential cumulative impact of experiencing multiple types of DFV, variance-weighted least-squares test for linear trend of violence victimisation (range 0–3 violence types) was performed. Data was imported, cleaned and coded using SPSS (version 25) [31] and analysed with STATA (version 15) [32]. Research ethics approval was granted by (Ethics ID: 1953826).

## Results

Of the 10,674 women nurses who completed a survey in the parent study, the response rate was 15.2% of all who had been sent an invitation email and 38.4% of those who had opened that email. Of the 5,982 participants who had experienced one or more types of interpersonal violence, most were born in Australia (78.0%), had median a age of 52 years, were living with a male partner (65.8%), and children (55.9%), and were working in a public hospital less than 35 h a week (67.4%) (Table 2).

**Table 2:** Demographic characteristics of participants (*n*, %)

Characteristic	All participants	12-month IPV	IPV longer than 12-months ago since sixteen years ^a^	Both IPV & child abuse	Both IPV & non-partner adult sexual assault	IPV, non-partner adult sexual assault & child abuse	ANMF member population ^b^	ABS PSS population % ^c^
	*N* = 10,629	*N* = 1,540	*N* = 2,515	*N* = 2,107	*N* = 1,080	*N* = 748	*N* = 79,264	*N* = 15,589
**Age (years)**	(*n* = 10,629)	(*n* = 1,529)	(*n* = 2,501)	(*n* = 2,099)	(*n* = 1,074)	(*n* = 744)		
< 30	1,109 (10.5)	194 (12.7)	171 (6.8)	164 (7.8)	104 (9.7)	58 (7.8)	16,098 (18.5)	15.3
30–39	1,937 (18.4)	356 (23.3)	370 (14.8)	350 (16.7)	186 (17.3)	130 (17.5)	23,015 (26.4)	19.3
40–49	2,213 (21.0)	408 (26.7)	546 (21.8)	489 (23.3)	242 (22.5)	164 (22.0)	17,689 (20.3)	18.3
50–59	3,182 (30.2)	429 (28.1)	856 (34.2)	712 (33.9)	354 (32.8)	261 (35.1)	17,595 (20.2)	17.2
60–69	1,962 (18.7)	137 (8.9)	528 (21.1)	367 (17.5)	180 (16.8)	124 (16.7)	10,007 (11.5)	16.2
≥ 70	116 (1.2)	5 (0.4)	30 (1.2)	17 (0.8)	8 (0.7)	7 (0.9)	918 (1.1)	13.6
**Country of birth**	(*n* = 8,227)	(*n* = 1,311)	(*n* = 2,158)	(*n* = 2,007)	(*n* = 1,026)	(*n* = 712)		
Australia	6,380 (77.5)	1,064 (81.2)	1,767 (81.9)	1,629 (81.2)	881 (85.9)	602 (84.6)	**	70.5
English first language	7,300 (89.0)	1,176 (89.7)	2,021 (93.6)	1,834 (91.4)	964 (93.9)	665 (93.4)	**	**
**Aboriginal/Torres Strait Islander**	104 (1.3)	16 (1.2)	28 (1.3)	31 (1.5)	17 (1.7)	17 (2.4)	**	**
**Intimate relationship status** ^d^	(*n* = 9,497)	(*n* = 1,540)	(*n* = 2,515)	(*n* = 2,107)	(*n* = 1,080)	(*n* = 748)		
**In a current relationship**	6,827 (71.9)	1,357 (88.1)	1,448 (57.6)	1,471 (69.8)	755 (69.9)	522 (69.8)	**	57.1
**Relationship during past 12mths**	7,201 (75.8)	1,539 (99.9)	1,525 (60.6)	1,588 (75.4)	822 (76.1)	564 (75.4)	**	**
**Ever been in a relationship**	9,021 (95.0)	1,540 (100.0)	2,515 (100.0)	2,107 (100.0)	1,080 (100.0)	748 (100.0)	**	81.7
**Sex of current partner** ^e^	(*n* = 6,297)	(*n* = 1,299)	(*n* = 2,137)	(*n* = 2,000)	(*n* = 1,024)	(*n* = 708)		
Male	6,135 (97.4)	1,141 (87.8)	1,298 (60.7)	1,418 (70.9)	726 (70.9)	497 (70.2)	**	62.4
Female	158 (2.5)	34 (2.6)	39 (1.8)	45 (2.3)	22 (2.1)	16 (2.3)	**	Female: 0.3
Non-binary	4 (0.1)	2 (0.2)	1 (0.1)	3 (0.1)	1 (0.1)	1 (0.1)	**	**
**Current living situation**								
Living with partner (incl. married)	5,673 (90.1)	982 (75.3)	1,153 (53.9)	1,255 (62.8)	648 (63.3)	450 (63.6)	**	47.9
In a relationship, but not living with partner	514 (8.1)	150 (11.5)	148 (6.9)	166 (8.3)	84 (8.2)	53 (7.5)	**	**
Separated	306 (4.8)	86 (6.6)	130 (6.0)	131 (6.6)	56 (5.5)	44 (6.2)	**	4.9
Divorced	634 (10.1)	88 (7.6)	349 (16.3)	253 (12.7)	111 (10.8)	69 (9.7)	**	12.5
Widowed	209 (3.3)	11 (0.8)	80 (3.7)	53 (2.7)	20 (1.9)	16 (2.2)	**	6.6
Not in a relationship/single	1,093 (17.3)	76 (5.8)	390 (18.2)	261 (13.1)	159 (15.5)	117 (16.5)	**	42.9
**Children**	(*n* = 8,177)	(*n* = 1,305)	(*n =* 2,151)	(*n =* 1,994)	(*n* = 1,022)	(*n* = 708)		
No children	2,093 (25.6)	286 (21.9)	486 (22.6)	415 (20.8)	242 (23.7)	152 (21.5)	**	**
Currently pregnant	126 (1.5)	76 (5.8)	33 (1.5)	22 (1.1)	9 (0.9)	4 (0.6)	**	**
1 + children living at home	4,519 (55.2)	833 (63.8)	1,162 (54.0)	1,169 (58.6)	582 (56.9)	408 (57.6)	**	32.2
**Work hours p/wk**	(*n* = 8,004)	(*n* = 1,279)	(*n* = 2,110)	(*n* = 1,963)	(*n* = 1,004)	(*n* = 696)		
Part-time (< 35 h)	5,390 (67.3)	864 (67.6)	1,383 (65.5)	1,281 (65.3)	637 (63.5)	431 (61.9)	28.0	14.5
Full-time (35 + hours)	2,614 (32.7)	415 (32.4)	727 (34.5)	682 (34.7)	367 (36.6)	265 (38.1)	64.0	9.1

*Note* Denominators vary due to missing responses; base = all survey participants who responded; ** Comparable data either not collected or available; ^a^ Participants who had experienced IPV in the last 12-months not included; ^b^ ANMF (Vic Branch) October 2019 data; ^c^ 2016 PSS data provided by ABS (courtesy of Anthea Saflekos) 16 February 2021; ^d^ 513 participants were omitted as they had never been in a relationship; ^e^ 1,845 participants did not have a partner at the time of the survey

### Prevalence of polyvictimisation

**Fig. 1:**
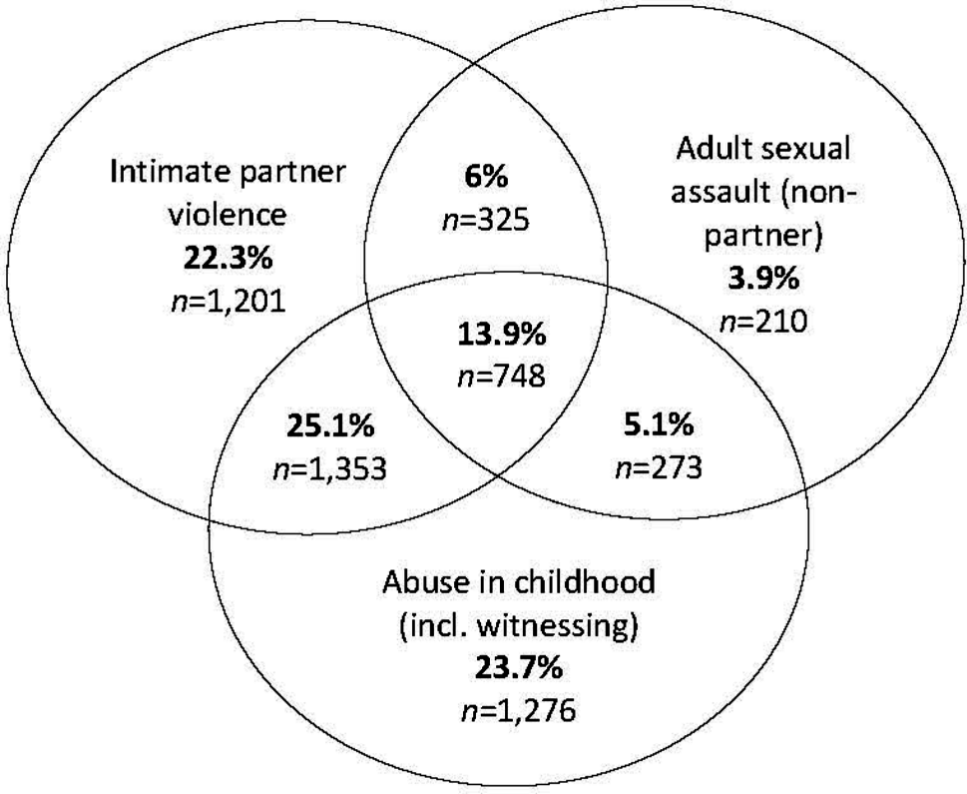
Overlap of IPV, non-partner adult sexual assault and child abuse among 5,386 nurse survivors

Approximately half (49.9%, 2,687/5,386) of nurse survivors had experienced a single type of violence, while the other half (50.1%, 2,699/5,386) had experienced more than one type of violence in different combinations (Fig. 1). More than one in ten (13.9%, 748/5,386) survivor nurses had experienced three types of violence (Fig. 1). The predicted odds that a nurse had experienced IPV was between two and three times higher for survivors of childhood abuse compared to nurses without child abuse (OR 2.7, 95% CI 2.4, 2.9). The odds that a nurse had experienced non-partner adult sexual assault were three and a half times higher for survivors of IPV compared with nurses who had not experienced that violence (OR 3.5, 95% CI 3.1, 3.9). Surviving child abuse more than trebled the adjusted odds of experiencing both IPV and non-partner sexual assault in adulthood (OR 3.4, 95% CI 3.0, 3.9).

### Health impacts of violence

Exposure to lifetime abuse was associated with an increased likelihood of reporting adverse health and well-being outcomes on all measures (Table 3). More than a third (35.9%) of polyvictimised survivors met the core criteria for generalised anxiety disorder [24] and nearly half (46.8%) for posttraumatic stress disorder [25] (Table 3). In general, the proportion of participants reporting health issues increased as the types of abuse they had experienced increased (Table 3).

**Table 3:** Proportion of nurse survivors currently experiencing health issues

Health and well-being measures	Participants not exposed to violence	Lifetime IPV survivors alone ^a^		IPV & child abuse (both) survivors ^b^		IPV, non-partner adult sexual assault & child abuse survivors (all) ^c^		Test for linear trend ^d^
	(*n* = 3,460)	(*n* = 1,201)	(*n* = 3,969)	(*n* = 1,353)	(*n* = 7,066)	(*n* = 748)	(*n* = 8,700)	*P-v*alue
**General health**	*n* (%)	*n* (%)	OR (95% CI)	*n* (%)	OR (95% CI)	*n* (%)	OR (95% CI)	
Poor physical health ^e^	39 (1.1)	25 (2.1)	2.5 (1.4, 4.5)	38 (2.8)	2.1 (1.4, 3.1)	30 (4.0)	2.3 (1.5, 3.4)	0.000
Hazardous alcohol consumption ^f^	306 (8.8)	174 (14.5)	1.8 (1.4, 2.2)	228 (16.9)	1.7 (1.5, 2.0)	146 (19.5)	1.8 (1.5, 2.2)	0.000
**Psychological health**								
Anxiety ^g^	534 (15.5)	287 (23.9)	1.9 (1.6, 2.2)	407 (30.1)	2.0 (1.8, 2.3)	268 (35.9)	2.1 (1.8, 2.5)	0.000
Depression ^h^	348 (10.1)	200 (16.6)	2.1 (1.7, 2.5)	314 (23.2)	2.1 (1.8, 2.5)	211 (28.3)	2.2 (1.8, 2.6)	0.000
Posttraumatic stress ^i^	488 (14.1)	315 (26.3)	2.5 (2.1, 2.9)	526 (38.9)	2.8 (2.5, 3.2)	350 (46.9)	2.9 (2.5, 3.3)	0.000
**Well-being impacts**								
Diminished resilience ^j^	240 (8.1)	113 (9.9)	1.4 (1.1, 1.7)	154 (11.8)	1.4 (1.1, 1.7)	98 (13.7)	1.5 (1.2, 1.8)	0.000
Lack of family/friends support ^k^	345 (11.6)	223 (19.6)	1.9 (1.6, 2.3)	419 (32.1)	2.6 (2.3, 3.0)	288 (40.2)	2.9 (2.4, 3.4)	0.000
Financial stress ^l^	609 (21.2)	404 (36.0)	2.1 (1.8, 2.4)	590 (46.6)	2.4 (2.1, 2.7)	373 (53.4)	2.5 (2.1, 2.9)	0.000

*Note* Denominators vary due to missing responses; base = all survey participants who responded; Reference category for each violence type was no exposure to that type ^a^ One type of DFV (not in combination with non-partner adult sexual assault or child abuse) ^b^ Two types of DFV ^c^ Three types of DFV ^d^ Variance-weighted least squares test for linear trend ^e^ SF-12 [29] ^f^ FAST [26] ^g h^ PHQ-4 [27]^i^ The Short Screening Scale for DSM-IV [25] ^j^ CD-RISC2 [28] ^k l^ Bespoke

### 12-month IPV

Compared to survivors who had not been exposed to IPV during the last 12-months, 12-month survivors reported worse health and well-being as lifetime polyvictimised survivors on all measures except for hazardous alcohol consumption (Table 4).

**Table 4:** Survivors of IPV during last 12 months: Health and well-being outcomes analysis

Health & well-being measure	Participants not exposed to IPV in the last 12 months	IPV in last 12 months ^a^	Effect estimate	*P*-value
	***n*** **(%)**	***n*** **(%)**	**OR (95% CI)**	
**General health**	(*n* = 5,444)	(*n* = 1,540)	(*n* = 6,510)	
Poor physical health ^b^	75 (1.4)	52 (3.4)	2.5 (1.7, 3.6)	0.000
Hazardous alcohol consumption ^c^	641 (11.8)	301 (19.6)	1.8 (1.6, 2.1)	0.000
**Psychological health**				
Anxiety ^d^	992 (18.2)	517 (33.6)	2.3 (2.0, 2.6)	0.000
Depression ^e^	664 (12.2)	393 (25.5)	2.5 (2.1, 2.8)	0.000
Posttraumatic stress ^f^	1,042 (19.2)	667 (43.3)	3.2 (2.8, 3.6)	0.000
**Well-being impacts**				
Diminished resilience ^g^	408 (8.2)	182 (13.8)	1.8 (1.5, 2.1)	0.000
Lack of family/friends support ^h^	722 (14.5)	475 (35.9)	3.3 (2.9, 3.8)	0.000
Financial stress ^i^	1,344 (27.6)	658 (50.9)	2.7 (2.4, 3.1)	0.000

*Note* Denominators vary due to missing responses; base = all survey participants who responded; Reference category was no exposure to IPV in the last 12 months; ^a^ Exposure to 12 month IPV measured via Composite Abuse Scale (CAS) ^b^ SF-12 [29] ^c^ FAST [26] ^d e^ PHQ-4 [27] ^f^ The Short Screening Scale for DSM-IV [25] ^g^ CD-RISC2 [28] ^h i^ Bespoke

## Discussion and implications

This is the first study to investigate experiences of DFV polyvictimisation and associations with health and well-being among nurses, midwives and carers. It adds to a growing body of research with community samples linking polyvictimisation with poor health and well-being [7, 9–12]. All three of our hypotheses were upheld. Hypothesis one: Participants who had experienced child abuse were more likely to have experienced IPV and non-partner adult sexual assault, indicating that the experience of child abuse may influence future abuse experiences. This is consistent with other research that abuse in childhood may predispose a life course effect [11, 33–36]. Hypothesis two: The frequency, proportion and odds of health and well-being issues increased as the types of violence to which a participant had been exposed increased, except for hazardous alcohol consumption, consistent with previous research in the general community [1, 9, 37]. Hypothesis three: survivors whose IPV had occurred in the last 12-months reported the poorest health and well-being, consistent with the minimal literature suggesting a temporal association between IPV and health [13].

Findings indicate that polyvictimisation and health challenges are a heavy burden on the shoulders of nurses, midwives and carers who work at the frontline of identifying and responding to DFV in our community [38]. Yet, research has not established specific recovery and healing-orientated interventions for health professional survivors [21, 38]. The imperative for interventions at work is further supported by evidence about the employment conditions of many nurses. While a history of DFV in the lives of health professionals may be an enabler to good clinical care of survivor patients [39], caring for patients may inevitably bring up distressing or disturbing reminders of nurses’ own trauma [40]. Further, all health professionals are at risk of accumulating a vicarious/secondary trauma response (sometimes called ‘Compassion fatigue’ or ‘burnout’) resultant from exposure to stories and images of the abuse of others [41–43]. Research suggests that vicarious trauma responses may be hastened or heightened for people whose personal lives have included trauma [40]. Adding to this trauma load: nurses are a highly gendered healthcare workforce commonly exposed to aggression and sexual harassment from patients and colleagues [44, 45]. Vicarious trauma can affect many spheres of a sufferers’ life, symptoms often mirror primary traumatic stress responses [41, 46]. For health professional sufferers of primary and/or vicarious trauma, both their inner world and their world at work is likely to be significantly impacted [46]. There is a strong rationale for supporting health professionals as they care for the community through targeted interventions that both delay the onset of trauma responses and treat their emergence.

### Strengths and limitations

Strengths of this study include the large sample size of health professionals [19] and the range of validated measures used to investigate DFV [23, 24], health and well-being [25–29]. Limitations of this study include the response rate, which, while comparable to similar studies, raises the possibility that non-respondents may have differed from respondents in ways that affected our conclusions [16, 47–50]. This survey asked about DFV across the life course; however, participants may have experienced other traumatic life events that they were not asked about, potentially confounding this study’s results. Multicollinearity between predictor variables can produce large standard errors in logistic regression and may have been an issue in this analysis [51]. However, multicollinearity diagnostics were performed (including checking the individual coefficients, correlation table and variance inflation factor) and did not indicate an association of concern between the predictor variables (IPV, non-partner adult sexual assault, child abuse), perhaps because of the large sample size [51]. A final note of caution: given the data in this study was cross-sectional, causal inferences cannot be made between DFV, health and well-being [12].

### Implications

Future research should investigate effective, accessible and economical recovery approaches for survivor nurses and other health professionals. Survivor health professionals may face barriers to accessing professional DFV support in the community for many reasons, including, shame and embarrassment [52], fear that their professional regulator may be informed [21, 38, 53] and beliefs that they are less ‘deserving’ of professional support than others [53]. Recovery from DFV is not an individual-level responsibility only, workforce well-being is the remit of healthcare organisations [46, 54]. With vision, leadership and investment, organisations can support and strengthen the recovery journeys and clinical care capacity of their survivor workforce [38, 55, 56]. A useful framework to underpin this cultural change is trauma and violence-informed care [57–59]. Building the capacity of hospitals and healthcare services to become trauma and violence-informed organisations that more effectively heal patients and better meet the needs and potential of staff, is critical [60].

## Conclusion

The findings of this study suggest a cumulative, temporal and injurious life course effect of abuse. The community stands on the shoulders of nurses, midwives and carers; to support recovery and healing of the survivor healthcare workforce and address the barriers survivors may face accessing mainstream community support, research into accessible and effective recovery-orientated workplace interventions is needed.

## Data Availability

At present, the data and materials (survey) are not publicly available but can be obtained from the authors upon reasonable request; contact author EM. The Composite Abuse Scale and many of the other measures in Table 1 are publicly available [23, 25–29].

## Abbreviations

CAS: Composite Abuse Scale
CI: Confidence interval
DFV: Domestic and family violence
IPV: Intimate partner violence
OR: Odds ratio
PSS: Personal safety survey
SCA: Severe combined abuse
